# The Serum Treatment of Puerperal Fever

**Published:** 1896-11-07

**Authors:** 


### THE SERUM TREATMENT OF PUERPERAL FEVER.

A large and a growing experience seems to show that puerperal septicæmia is a preventable disease, while all experience teaches that it is due to a local infection, which in many cases is a continuously acting one having its seat within the uterus.

No possible means of general treatment of the blood poisoning, the septicæmic side of the difficulty, can ever take away the necessity for local treatment of the condition, where such exists, by which the poisoning is being kept up; and certainly no promise of cure by serum treatment can lessen by a hairsbreadth the obligation which lies on every accoucheur of conducting his manipulations in such a fashion as to minimise the chance of infection to his patient. But in actual practice, when we see a case of puerperal septicæmia, the mischief has already taken place; and while doing everything to prevent the continuance of the infection, where a continuously acting cause exists, we must welcome thankfully anything which will enable us to neutralise such septicæmia as has already taken place. Cases may even occur in which this latter may constitute the whole disease, but, as a rule, both conditions are in operation at the same time. In considering, then, the uses of the anti-streptococcic serum which is now available for the treatment of puerperal septicæmia, it will not do to overlook the fact that this disease, or symptoms which cannot be differentiated from it, is at times set up by an infection with other microbes, and that a serum which may be effectual against the streptococcus pyogenes will have no antitoxic influence against infection by gonococci or staphylococci. A bacteriological examination of the discharges may, therefore, be of great service in the selection of cases; but if, with larger experience, the treatment by "serum" should prove as useful as from recent reports seems to be the case, we should hardly refuse a patient the chance of benefit from its use on the strength of a negative result from such an examination, for the organisms might well exist in a virulent form, and yet be cut off from all communication with the vagina. In the great majority of cases, however, the disease is due to the streptococcus pyogenes, and where that is the case it seems clear that the use of antitoxic serum is, at the least, able to hold existing infection in check, and thus to give time during which infective surfaces may be cleansed; while, if the infection has arisen from a part, say a laceration, which has already become clean, the serum may actually cure the disease and land the patient again in safety. It is impossible to admit the efficacy of anti-diphtheritic serum without admitting also that against other diseases similar antidotes will be discovered. While, then, in every case of puerperal septicæmia we do all that is possible to remove all causes of infection, we shall do well also to employ the anti-streptococcic serum which is now at our disposal, seeing that both theoretical teaching and practical experience point to its utility as, at the least, a means of holding in check, and perhaps neutralising, the consequences of some forms of infection until its sources are done away with.

